# LGBT+ partner bereavement and appraisal of the Acceptance-Disclosure Model of LGBT+ bereavement: A qualitative interview study

**DOI:** 10.1177/02692163221138620

**Published:** 2022-11-25

**Authors:** Katherine Bristowe, Liadh Timmins, Debbie Braybrook, Steve Marshall, Alexandra Pitman, Katherine Johnson, Elizabeth Day, Paul Clift, Ruth Rose, Deokhee Yi, Peihan Yu, Wei Gao, Anna Roach, Kathryn Almack, Michael King, Richard Harding

**Affiliations:** 1 Cicely Saunders Institute of Palliative Care, Policy & Rehabilitation, King’s College London, Bessemer Road, London, UK; 2 School of Psychology, Swansea University, Wales, UK; 3 King’s College Hospital NHS Foundation Trust, Denmark Hill, London, UK; 4 Division of Psychiatry, University College London, London, UK; 5 Camden and Islington NHS Foundation Trust, St. Pancras Hospital, London, UK; 6 Social and Global Studies Centre, Royal Melbourne Institute of Technology, Melbourne, VIC, Australia; 7 Patient and Public Involvement, London, UK; 8 Great Ormond Street Institute of Child Health, Faculty of Population Health Sciences, London, UK; 9 School of Health and Social Work, University of Hertfordshire, Hatfield, Hertfordshire, UK

**Keywords:** Bereavement, LGBT, spouses, sexual and gender minority, qualitative research

## Abstract

**Background:**

Support from social networks is vital after the death of a partner. Lesbian, gay, bisexual and/or transgender (LGBT+) people can face disenfranchisement and isolation in bereavement. The Acceptance-Disclosure Model (of LGBT+ bereavement) posits that experiences are shaped by the extent to which individuals feel able to disclose their bereavement to others, and whether that loss is acknowledged appropriately.

**Aim:**

To explore LGBT+ specific experiences of partner bereavement; determine decision-making processes regarding disclosure of relationships/identities; and appraise the Acceptance-Disclosure Model using primary qualitative data.

**Design:**

Exploratory in-depth qualitative interview study positioned within a social constructivist paradigm. Data were analysed using inductive and deductive reflexive thematic analysis.

**Setting/participants:**

21 LGBT+ people from across England bereaved of their civil partner/spouse.

**Results:**

Participants described LGBT+ specific stressors in bereavement: lack of recognition of their loss; inappropriate questioning; unwanted disclosure of gender history; and fears of discrimination when accessing support. Disclosure of LGBT+ identities varied across social networks. Some participants described hiding their identities and bereavement to preserve relationships, and challenging intersections between LGBT+ identities and other aspects of culture or self. These findings provide primary evidence to support the Acceptance-Disclosure Model.

**Conclusions:**

LGBT+ people face additional stressors in bereavement. Not all LGBT+ people want to talk directly about their relationships/identities. Sensitive exploration of support needs, aligned with preferences around disclosure of identities, can help foster trust. Five recommendations for inclusive practice are presented. Further research should consider whether the Acceptance-Disclosure Model has utility to explain bereavement experiences for other isolated or disenfranchised groups.

**What is already known about the topic?**

- After the death of a partner, support from social networks is vital to avoid adverse bereavement outcomes.
- LBGT+ people face isolation and disenfranchisement in bereavement due to fears and previous experiences of discrimination.
- The Acceptance-Disclosure Model of LGBT+ bereavement posits that experiences for LGBT+ people are shaped by their ability to disclose their bereavement to others, and whether that loss is acknowledged appropriately.

**What this paper adds?**

- Additional barriers and stressors in bereavement can limit access to essential support for LGBT+ people.
- LGBT+ people demonstrate agency in their decisions regarding disclosure of identities and relationships. The extent of disclosure often varies across their social networks.
- Challenges were described due to intersections between LGBT+ identities and other aspects of culture or identity. Some individuals chose to hide their relationship and bereavement in order to retain important relationships and avoid disenfranchisement.
- Five recommendations for inclusive care of LGBT+ people facing bereavement derived from the data are presented.

**Implications for practice, theory or policy**

- Direct questions about relationships and identities can feel confrontational for LGBT+ people due to fears and previous experiences of discrimination.
- Sensitive communication, and respecting autonomy regarding disclosure, can support person-centred care.
- Our findings support the Acceptance-Disclosure Model to explain experiences of LGBT+ bereavement. This model could also inform assessment and support strategies for other potentially isolated or disenfranchised groups.

## Background

The impact of bereavement is felt most heavily by those closest to the deceased,^1,2^ with primary caregivers at risk of poor bereavement outcomes.^3^ However, bereaved partners infrequently access health care^4^ despite increased odds of worsening or new physical illness^5^ and mortality.^6^ Access to support from social networks is vital to avoid adverse bereavement outcomes including psychosocial morbidity and prolonged grief symptoms,^7^ and to enable adaptation after the loss.^8^

Lesbian, gay, bisexual and transgender (LGBT+) people constitute minority groups with specific healthcare needs,^9^ including greater all-cause mortality^10^; increased risk of common mental disorders, substance misuse and suicidality^11–13^; more health risk behaviours^14,15^; and therefore increased risk of life-limiting illnesses.^16–18^ With higher rates of mental health conditions, LGBT+ people may have worse bereavement outcomes, particularly where compounded by increased isolation reducing essential social support. Indeed recent US research which mapped social networks for older LGBT adults found that those with the most restricted social networks had poorer mental health.^19^ Similarly, in bereavement, LGBT adults who reported good social support, described more positive coping behaviours,^20^ highlighting the importance of informal support in bereavement.

Despite protection under UK law (Equality Act (2010), Health and Social Care Act (2012)), experiences of discrimination for LGBT+ people persist, including at the end of life,^17^ and into bereavement.^21,22^ Sensitive communication is therefore vital to build trusting relationships between LGBT+ people and their clinicians.^23,24^ Incorrect assumptions about relationships lead to disengagement and loss of trust, resulting in reluctance to access healthcare.^25,26^ Lack of acknowledgement of the depth of the relationship, or experiences of discrimination around the bereavement period, can have devastating implications for LGBT+ bereaved partners. Such disenfranchisement reduces their access to vital social support afforded during grieving.^27^

**Figure 1.**
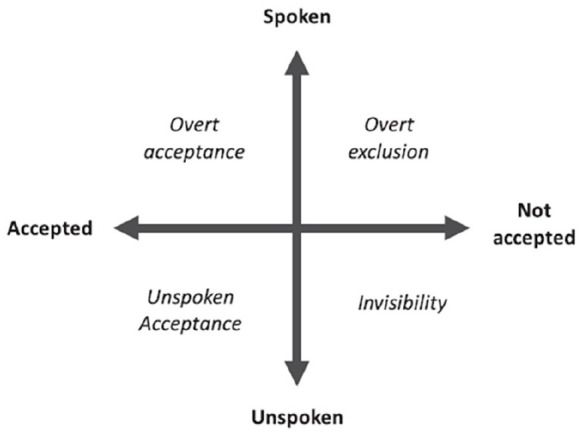
Acceptance-disclosure model of LGBT+ bereavement experiences.^21^

A recent systematic review describing the breadth of additional barriers and stressors for LGBT+ partners in bereavement^21^ informed the development of the Acceptance-Disclosure Model of LGBT+ bereavement (see Figure 1). This posits that the experience of bereaved LGBT+ people is shaped by the extent to which individuals feel able to disclose the nature of their relationship to others, and the degree to which that relationship is accepted. The model describes four domains of bereavement based on the four dimensions; spoken, unspoken, accepted, not accepted.

The domain within the model that an individual occupies is theorised to influence their bereavement experience and their ability to access support. If for example an individual occupies the domain of ‘overt acceptance’, their relationship is known and accepted by those around them, and therefore they are recognised as facing bereavement and will be able to access support. In contrast an individual occupying the domain of ‘invisibility’, has not felt able to share the nature of their relationship with those around them, and their grief will also remain invisible. Therefore their access to the grieving role, and the support afforded by that, would be limited. The domain of ‘unspoken acceptance’ represents those individuals who, for whatever reason, prefer not to directly explain the nature of their relationship, however they would assume that those around them recognise the depth of the relationship (also called tacit acknowledgement^28^). The risk for these individuals is that the relationship may be misunderstood and therefore they may not receive adequate support. Finally, those in the domain of ‘overt exclusion’ have felt able to share the nature of their relationship, however this has been met with rejection and exclusion from those around them, adding markedly to the trauma of their loss.

Primary quantitative data provides preliminary support for this model, as loneliness, social support and caregiver burden are associated with the intensity of grief, and psychological symptoms are greater for bereaved same-gender partners.^29^ The present study aimed to (1) investigate LGBT+ specific experiences of partner bereavement; (2) to examine decision-making processes in relation to disclosure of relationships and identities; and (3) appraise the Acceptance-Disclosure Model of LGBT+ bereavement using primary qualitative data.

## Methods

### Design

Our research team comprises mental health and palliative care clinicians, qualitative methodologists, social scientists, psychologists, health services researchers, researchers experienced in LGBT+ health research, and LGBT+ community members. This exploratory qualitative interview study is positioned within a social constructivist paradigm, which suggests that learning and knowledge develop from an individual’s interactions with their culture and society. This qualitative study was nested in a larger population-based study (ACCESSCare-B) incorporating a national cross-sectional survey of bereaved same-gender and different-gender partners^29^ 6–10 months post bereavement.

### Participants and settings

Individuals who had registered the death of a civil partner or spouse in England and Wales were invited to participate in the ACCESSCare-B survey.^29^ All survey participants were asked whether they would be willing to be contacted for a subsequent one-to-one qualitative interview. A comprehensive demographic variables section was included within the ACCESSCare-B survey. This enabled the research team to purposively sample individuals who had consented to be contacted for interview and had reported the death of a same-gender partner, by the following criteria (of both participant and decedent), to maximise the diversity of the sample and increase transferability of the findings: gender, age, sexual orientation, gender history and ethnicity.

### Recruitment

Participants were contacted by the study researcher (LT), who explained the interviews in more detail. Participants were offered a choice of interview modality (in person or telephone) and location (their own home, a university building, another setting). All participants gave written informed consent prior to the interview. No participants were known to the study team prior to recruitment. No incentives or payments were provided to participants in this study, however all participants were given a leaflet on bereavement and a signposting resource for bereavement support services.

### Interview data collection

All interviews were conducted by the study postdoctoral researcher (LT), a psychologist new to qualitative interviewing, but with training in person-centred counselling and experience as a helpline volunteer. They were supervised by the study lead (KB), a qualitative methodologist. The interview topic guide was devised by KB, LT and RH and refined through discussion with the study research team including LGBT+ community members (see Supplemental Materials for a copy of the interview topic guide). Each interview commenced with demographic questions about the participant and their deceased partner, to allow individuals to describe these important elements of self in their own terms without the restrictions afforded by a survey. They then explored the illness experience, and the participant’s involvement in their partner’s care and care planning. Subsequent questions explored preferences regarding disclosing relationships, sexual orientation and/or gender identity to others in their social networks and to health care professionals. Questions then explored the partner’s death, funeral and sources of support both pre-and post-bereavement. The interviews closed with an opportunity to share recommendations for practice. After each interview a reflective diary was completed by the interviewer (LT), and discussed with the study lead (KB) and other members of the research team. The diary included emergent themes related to the aims of the study, commonalities and differences in experiences described compared to preceding interviews, and any environmental factors that may have influenced the conduct of the interview. These emergent themes were presented, discussed and revised with the whole steering group, including LGBT+ community members. An iterative process of discussion of the reflective diary, alongside the study aims, informed the decision to stop recruitment, and move to analysis. Recruitment continued until saturation was indicated and no new themes related to the study aims were being identified.

### Analysis

Interviews were audio recorded and transcribed verbatim. Analysis was conducted in two phases supported by NVivo qualitative data analysis software. First, an inductive reflexive thematic analysis^30^ was conducted to explore bereavement experiences and sources of support. Analysis followed six stages: familiarisation, generating initial codes, generating themes, reviewing potential themes, defining and naming themes and reporting. Analysis was led by KB, but interpretation was collaborative and iterative throughout the stages of the analysis, drawing on the diverse perspectives and experiences of those within the research group. Descriptions of themes and coded extracts were shared with the research team, including LGBT+ community members, to comment on and revise interpretations. A secondary deductive reflexive thematic analysis was conducted to appraise the extent to which the data supported the Acceptance-Disclosure Model of LGBT+ bereavement.^21^ This stage of the analysis included a return to the initial codes, and generation of themes within the four domains of the model.

The present study is reported in line with the COREQ (consolidated criteria for reporting qualitative research) guidance.^31^ Labels for verbatim quotes state gender modality only where this was shared during the interview. All names were replaced with culturally appropriate pseudonyms.

## Results

### Participants

Twenty-one individuals were interviewed (October 2018–September 2019) who were bereaved of a same-gender civil partner (*n* = 17) or spouse (*n* = 4). Nine identified as women, eleven as men and one as non-binary. Although not explicitly asked about gender modality (how a person’s gender identity stands in relation to their gender assigned at birth^32^), two participants shared that they were transgender, and three participants shared that their deceased partners were transgender. The remaining participants did not share information about their own or their partner’s gender modality during the interviews. Most participants described their sexual orientation as gay, lesbian, bisexual, or same-sex attracted (*n* = 18), one as heterosexual, two preferred not to say and one did not feel that the label was important. Six of the participants were from minority ethnic communities. The median age of participants was 57 (range 37–85), and median relationship duration was 19 years (range 7–61 years). Participants were recruited from across England, and the median interview duration was 79 min (range 38–194) (Table 1).

**Table 1.** Characteristics of interview participants, deceased partners, relationships and interviews.

Characteristics		Interview participants	Deceased partners
Gender	Woman	9	8
	Man	11	12
	Non-binary	1	1
Gender history	Cisgender	19	18
	Transgender	2	3
Ethnicity	White British, white Irish or white other	15	19
	Asian	2	0
	Multiple ethnic background	1	0
	Black African	0	1
	Black other	1	0
	Other ethnic group	2	1
Sexual orientation	Lesbian	2	3
	Gay	11	11
	Bisexual	4	3
	Same-sex attracted	1	1
	Heterosexual	1	1
	Not sure	0	1
	Prefer not to say	1	1
	Not important	1	0
Age	Median (range)	57 (37–85)	61 (44–89)
Religion or religious background	Agnostic	2	1
	Atheist	2	2
	Christian	5	8
	Hindu	1	0
	Muslim	1	0
	Religious (not specified)	1	1
	Not stated	9	8
	None	0	1
Relationship status	Married	4	
	Civil partnered	17	
Median total relationship duration (range)		19 years (7–61)	
Living situation	Living together	18	
	In shared accommodation with others or care settings	3	
Cause of death of partner	Cancer	8	
	Non-cancer (cardiac arrest, brain haemorrhage, brain aneurysm, aortic dissection, dementia, heart failure, Chronic Obstructive Pulmonary Disease, pulmonary fibrosis, Motor Neurone Disease, multi-system atrophy)	13	
Geographical location	North of England	5	
	Midlands	3	
	Greater London	6	
	South of England	6	
	Unknown	1	
Interview location	Participant’s home	8	
	At university site	3	
	Other	1	
	Via telephone	9	
Median interview duration (range)		79 min (38–194)	

## Findings

Participants’ bereavement experiences are presented below. Part 1 focuses on descriptions of the emotional and social response to bereavement, which we conceptualised as falling into two categories; those that accorded with universal experiences of partner loss (as derived from other published literature), and those that appeared to be specific to LGBT+ groups. The second part presents the appraisal of the Acceptance-Disclosure Model of LGBT+ bereavement.

### Part 1: Bereavement experiences

#### a. Perceived universality of emotional and social response to partner bereavement

Participants were asked whether they felt their bereavement experience differed in some way because of their LGBT+ identities. Many felt there was no difference, and that the devastation at the loss of a partner was universal.

*‘I just miss his company, [. . .] loving him and him loving me. I think that’s the same with a straight couple as well [. . .] you miss the company of the person first and foremost’.* Jamal, gay man, in his 40s

For some individuals there was a degree of acceptance of their grief. This was ‘another phase’ of their life, they accepted that ‘change is normal’, and ‘nothing is forever’.

*‘People find that, that they look for things staying the same as being the normal [. . .] things are always going to change [. . .] you’ve got to be able to[. . .] accept that’.* Robin, gay man in his 60s

However, others described being struck by the enormity of grief, having lost ‘the unit’, and a part of themselves too.

*‘It’s very different [long pause] [. . .] It’s, it’s more emotional [. . .] it’s literally yourself, you. . .you lose a part of yourself and [. . .] I didn’t experience that before’.* Alexander, gay man in his 40s

They talked of loneliness, but also a profound sense of being alone. Everything in their life was now different, which was hard to reconcile.

*‘I don’t think you’re ever prepared to feel so utterly destroyed by bereavement. The thing is so monumental, and you have to [. . .] learn how to do everything again [. . .] you have to learn how to breathe again and how to eat again and how to walk again [. . .] when you’re in a partnership with somebody, you walk a certain way or I always walk on this side because there was a hand I’d like to hold [. . .] and everything is different. [. . .] underneath it’s a great sea of lurking dark thing that is your bereavement, that is your loss and initially it takes over and there is nothing else’.* Louise, bisexual woman in her 30s

This was compounded by the ebbing away of support from friends and family only months after their bereavement, just when they needed it the most.

*‘The first three months afterwards, erm were the worst because everything goes quiet. [. . .] You suddenly go from a huge busy time to absolute stillness and silence and you feel very alone and that’s probably the time where you actually need people to come round’*. Rachel, lesbian woman in her 50s

Some participants struggled to engage emotionally with their loss, feeling ‘numb’ or ‘empty’, while others responded by hiding their grief, as they felt this was what people preferred.

*‘I’ve learnt to do what I call ‘the monkey act’ which is you can perform for several hours without breaking down in tears [. . .] it does make it easier for everyone and that is important because if they see you crying at a (party), it’s a stress for everyone [. . .] Everyone close to me knows how much I’m suffering. when I’m in public or at an event then I can control it for a prolonged period of time. [. . .] And that is a relief for everyone concerned, including me, because it means it doesn’t draw attention to me’.* Anthony, gay man in his 50s

Expectations of how to manage grief were touched on by many participants. They talked about people around them not wanting to discuss the death directly, favouring euphemisms instead and avoiding mentioning the deceased partner by name.

*‘What annoys me [. . .] when we talk about someone dying, they’ve passed away [. . .] I felt it important to say that he had died. [. . .] the fact that I actually saw him die in front of my eyes, you know in front of me, err and then the paramedics were there for 45 erm [. . .long pause] I, I think it was important, erm grieving process to mention that’.* Robin, gay man in his 60s

In the first year post bereavement, the grief would at times hit them ‘out of the blue’. Sometimes individuals were unable to control the urge to cry and needed people around them who would tolerate that.

*‘I found Valentine’s Day a nightmare [. . .] I’ve always done cards and I bought her some flowers and whatever and so I didn’t go near the shops cos I thought it’ll be full of red roses. And I’m going into [the supermarket] two days later and they’re selling off all the red roses- I was devastated. [. . .] But, now, I’ve done all the first anniversaries of everything [. . .] and it’s been quite intensive cos they came together [. . .] But if you don’t do it, it will get you in the end. It’s like any problem, you can’t put the lid on it- one day the lid will blow off’*. Patricia, woman in her 70s bereaved of a female partner

Participants described two different experiences of social connectedness following their bereavement; either gravitating towards or away from others. Those who gravitated towards others talked about the importance of company, and recognition of their need for support.

*‘They’ve been lovely. Very much my neighbours are very supportive and they’re always there when I need it. [. . .] When I’m away they take the dog for a walk, they let me go out for dinner or invite me up for a drink [. . .] They’re very nice and supportive’* Jose, gay man in his 50s

However, companionship was sometimes at the cost of a sense of guilt for continuing life without their partner.

*‘One neighbour who lost her husband a couple of years back and I have formed quite a good liaison [. . .] We get on extremely well you know, so that’s been good. . . You sometimes feel guilty you’re not feeling immense grief, err that you’re actually doing things to enjoy yourself when you shouldn’t be enjoying yourself’* Eric, gay man in his 80s

For those who spent much time alone, some talked of a need for this as self-preservation, while others described a process of evolution, as they learnt that time alone was what they needed to grieve, regain control and heal.

*‘[Partner] said you’re gonna have to learn to be strong, and this is what he meant I think. You have to say no if you think the thing you’re being asked to do will cause you more damage than good. To begin with, I think I was so lost that [. . .] I was saying yes to everything [. . .] Since I’ve learnt the power of no, [. . .] I don’t feel better, how could I, I have more control over my own environment. So being at home alone is often succour [. . .] I appreciate my own space’.* Anthony, gay man in his 50s

Others talked about feeling disconnected and isolated in their grief. They described lacking people they could relate to about their bereavement, and concerns about overburdening friends and family.

*‘I find myself isolating myself because I get upset with other people and I don’t want to hurt them, if that makes sense. [. . .] My sadness is contagious. [. . .] I’ve seen people that are having a really good day and a laugh and they’ll say to me ‘How are you doing?’ and if I’m having a bad day and I, and I tell them and then I can see they just kind of shrink into themselves and I’ve then ruined their day, so this, I don’t want to do that to people’.* Louise, bisexual woman in her 30s

#### b. LGBT+ specific experiences

Participants also described challenges in their bereavement that appeared to be LGBT+ specific, such as conflicts and lack of acceptance of their LGBT+ identities from family members, and subsequent lack of support.

*‘My mum and dad found it quite difficult to accept that myself and [Partner] were together [. . .] I think they would have been more supportive if [Partner] had been a man [. . .] Even though they knew she was unwell, they didn’t really provide an awful lot of emotional support and they didn’t really understand or accept that we were going through a difficult time’.* Aoife, bisexual woman in her 40s

For others, challenges included financial and legal conflicts with family, particularly regarding capacity, power of attorney and wills, despite being married or civil partnered.

*‘At one point he was bundled up in the middle of the night by his mum and sister and transferred to his sister’s house [..] He resisted at first, but I think that night he was really unwell [. . .], so he didn’t put up a fight [. . .] Once I knew I just went in there ‘Look I don’t want to make a fuss because we are all here for [Partner] but do not do that kind of thing again. You know he’s at the best place’ [. . .] I promised myself until he is laid to rest, I don’t want to kick up a fuss with the family at all’*. Jamal, gay man in his 40s

There were also challenges for some individuals at the intersection between their LGBT+ identities and other identities, such as age and ethnicity. Some who had experienced bereavement at a relatively young age described intrusive questioning, or individuals minimising their loss. Questions tended to relate to finding a new partner; a question they felt would not be asked of heterosexual bereaved partners.

*‘I do have the odd person saying to me [. . .] ‘You’re very very young you know. You could find somebody else’ [. . .] I tend to shut that down very, very quickly. Erm because I have umm, am not in any place to even think about those kinds of things [. . .] whereas my friends in the LGBT community would never dream of asking me that’.* Louise, bisexual woman in her 30s

Others described incorrect assumptions about the nature of their relationship due to an age difference, which they felt would never be made of heterosexual couples.

*‘How’s your mum?’ or ‘Oh you’ve bought your daughter with you’. And they just automatically do that. I mean even when the ambulance brought her home at the end, they said ‘Oh hello, is this your Mum?’. they’d all apologise. [. . .] And I just used to think ‘Come on you’re a professional, you shouldn’t make that basic mistake’.’* Caroline, gay woman in her 60s

For others, stressors related to tensions with family members with a strong faith, for whom same-gender relationships were not acceptable.

*‘I mean the person who funnily enough has been least caring is my sister [. . .] I don’t think at any time has she asked me how I, how am I getting on, which is quite strange really. I think she assumes a gay relationship is not quite the same thing as a heterosexual relationship. [. . .] I think my brother-in-law disapproved. He was quite religious and so I always had a fairly stiff relationship with him’.* Eric, gay man in his 80s

Participants also described challenges regarding their cultural background when from countries that criminalise or stigmatise LGBT+ people. They could not disclose their relationships or bereavement with family members for fear of losing their relationship with them, or putting their family at risk of harm.

*‘No one knew in Iran that I’m gay [. . .] I didn’t want anyone to know, erm about me, because not only, not only is it erm, a crime in Iran to be gay, but also it’s a big shame or you don’t know how people react. Some people, they might be supportive, they might be open-minded, but my concern is more, it’s erm, it’s the shame that it brings [. . .] to my family. [. . .] If it was for me alone it would have been okay [. . .] So, I was very careful and I’m still very careful’*. Jason, gay man in his 40s

There were also specific challenges described relating to LGBT+ identities themselves. For individuals for whom their deceased partner had been their first same-gender partner, the bereavement led them to question themselves and their identity despite not feeling ready for another relationship.

*‘I questioned who I am, what I am, what I want. Because the only time I have been truly happy in (my life), was those 12 or 11 years that I was with [Partner]. A woman made me truly happy and it’s the only time that I’ve truly been loved [. . .] I would love to find somebody, umm that I can share my life with and feel comfortable with and understood me [. . .] If a a man came along [. . .] or whatever, would I, you know? So [. . .] sexual orientation after [Partner], it’s, it’s making me question myself’.* Rachel, lesbian woman in her 50s

Specific challenges were also shared in relation to gender identity. Participants described universally respectful and caring interactions with funeral providers, however these could be undermined by bureaucratic requirements to use the individual’s birth name on official documentation.

‘*She said that they would give him a shave and I said ‘no’ [. . .] She used the correct pronouns and she said, ‘but we, we shave all [. . .] the men when we prepare’ and I said, ‘but you can’t [. . .] because he’s only just started getting whiskers and if you take them away I’m gonna cry’ and so she left them [. . .] she said ‘this is what you’ve said and we’re gonna do the best that we can [. . .] On his casket [. . .] they were like ‘Look we have to put the name on his birth certificate’ and I was like ‘but that’s not’ [long pause] (crying) ‘that’s not his name’ [long pause] [. . .] They were very good about it and in the end [. . .] she did two plaques for me. One with his birth name, so they could identify the casket itself and then just below that she put another one, with his chosen name, which is his real name, on it, so that they didn’t have to follow it completely because I couldn’t bear that. Every time I had to scan something that has his birth name on it [long pause] that was horrible because that’s not who he was’*. Louise, bisexual woman in her 30s

Seeing their partner being immortalised as their birth name was a cause of significant distress for grieving partners. Similarly, another participant bereaved of a transgender partner described her partner’s gender history being disclosed by the cause of death (prostate cancer) on the death certificate.

*‘The certificate had got prostate cancer on it [. . .] She said to me, ‘Look I’m sorry but that’s the law’ [. . .] But that really sort of knocked me sideways and of course I had to go straight to the registrar’s to register the death [. . .] and obviously he can’t change the death certificate and he has to copy exactly what it, what it is onto the register and then you get the certificate which is an extract of that. So that’s now a public record that anybody can see, so that really pisses me off’.* Eileen, bisexual woman in her 70s

In contrast, another participant bereaved of a transgender partner described the importance of talking about all of her partner’s life and gender history during her funeral as it improved understanding about the depth and nature of their relationship.

*So, there was a bit before I met her and then the other forty years. It was sort of half as, before transition and half after transition [. . .] One of my brothers afterwards [. . .] he said he [. . .] hadn’t really fully understood [. . .] In the eulogy [. . .] I said like ‘Now most of you know that [Partner] was trans. . .transgender’ and then I sort of thought, well you know just that one [. . .] quite a sort of factual sentence [. . .] there was lots and lots hidden behind that because [. . .] she struggled with her identity and who she was [. . .] particularly during the transition years [. . .] [My brother] sort of apologised and said ‘I’m sorry, I didn’t understand it’.* Sally, heterosexual woman in her 60s

There were also LGBT+ specific considerations in relation to sources of support. Participants described fragile relationships with family members, but also how their lesser likelihood of having biological children resulted in many LGBT+ people having less support.

*‘I think, gay people tend to be more isolated [. . .] I don’t have family around, I don’t have kids, I don’t have anyone. So, like me [. . .] they’re in the same situation [. . .] I’m on my own everyday’.* Jose, gay man in his 50s

For many, LGBT+ friends provided a unique source of support. Shared experiences and history provided a closeness and an understanding of what they were going through.

*‘You’re probably able to open up to them more [. . .], gay friends than straight friends [. . .] You don’t have to explain as much to gay people, because they’ve been in the same situations and erm [long pause] or they might be in the same situation of losing their partner’.* Robin, gay man in his 60s

Few had accessed professional bereavement support. For some, reluctance to access community support stemmed from fears of discrimination.

*‘I have such limited contacts outside of the health system so I wouldn’t know, if I had gone to church or somewhere else how would they [have] treated me’.* Kamal, gay man in his 40s

Similar concerns were also raised in relation to health and social care services, with concerns expressed about the variability in treatment they might receive from different individuals.

*‘It’s a bit like the trying to find a care provider [. . .] You don’t actually know who you’re gonna be dealing with as an individual. I don’t know anything really about these organisations. You would hope that they were good on equalities, but you don’t really know. And in the end it comes down to an individual person doesn’t it? Not the organisation’*. Eileen, bisexual woman in her 70s

Others saw official environments and services (bound by equality legislation in the UK) as affording protection and giving them the confidence to share their identity. This was in contrast to negative experiences in public spaces.

*‘If I was out in public we were always a little bit more circumspect [. . .] So we wouldn’t hold hands in the street, we wouldn’t kiss in public [. . .] If I’m with officials, in any official environment now, I don’t care and I will be out there knowing full well that I’m protected. I’m not quite so cocky if I was in a gang of football fans’.* Anthony, gay man in his 50s

### Part 2: Acceptance-Disclosure Model of LGBT+ Bereavement^21^

In the second part of our analysis we applied a more deductive analytic approach to appraise the extent to which the data supported the four domains of the Acceptance-Disclosure Model of LGBT+ bereavement^21^. We focussed on data that captured participants’ views, experiences and preferences related to disclosure of their relationships and identities and the reasons behind these decisions. We were interested in whether they felt able to disclose the nature of their relationship to those around them (spoken vs unspoken), relating this to the degree to which that relationship felt recognised (accepted vs not accepted).

Participants described experiences which support the four domains of the model. However, they also highlighted a degree of autonomy and agency in these experiences, which had not emerged so strongly from the initial modelling of the literature. Generally, the data demonstrated varying degrees of agency in decisions to disclose, ranging from actively choosing their level of disclosure, through to this being elicited passively. As this agency has been demonstrated in relation to the position an individual occupies in the model, we have described this in terms of active positioning and passive positioning. Often participants occupied more than one domain concurrently, disclosing their relationship openly in some settings, while adopting a more unspoken or invisible position in others. The findings below are structured around the four domains of the model.

#### a. Overt acceptance

Participants described achieving overt acceptance both actively and passively.

*Active positioning*: The positive impact of overt acceptance was confirmed by the interview findings, with individuals describing how feeling able to talk about their bereavement enabled support from those around them.

*‘People in our surroundings [. . .] knew that erm, we were in a relationship and got civil partnered [. . .] I don’t think it was any different from [. . .] loss of a heterosexual partner. It would be more difficult if [. . .] they kept the relationship secret while they were living [. . .] What do you do when your partner you’ve felt a lot about dies? Because perhaps you feel you can’t reveal how you feel, because you’re revealing the relationship [. . .] It’s a lot harder for somebody in that situation than somebody in my situation’.* Robin, gay man in his 60s

Participants talked about taking an active role in achieving overt acceptance pre-bereavement so that professionals would know who mattered to their partner.

*‘He’s my partner and we’re in a civil partnership’, I don’t think I’ve ever used the term ‘We’re gay’, I think it was just always ‘He’s my partner’ and that tells you what you need to know. [. . .] I don’t, wouldn’t have any problem if someone said to me ‘Oh are you gay?’ erm, but it’s implied with what I just said and I think it’s the nature of my relationship with them that is the important fact and not the fact that we’re gay. It’s that, the trauma, it’s the relationship and what’s happened to him that matters and I’m not seeking counsel because I’m a gay man.’* Stuart, gay man in his 40s

Some talked about using their legal status (e.g. ‘This is my civil partner’) to achieve that, and to ensure they were involved in decision making.

*‘I mean the reason we did our civil partnership was really for the legality of it that would in law, make me next of kin. That was the driving force for it, not because we wanted to shout it from the rooftops [. . .] At the end of the day we’re just people and they accepted that we were in a relationship so we came as a duo and a package and that was the end of it’’*. Caroline, gay woman in her 60s

*Passive positioning*: Others in this domain did not show such a degree of agency, but instead effected disclosure more passively. This might be in response to questions about their relationship from the clinical team, and would not have been disclosed otherwise.

*‘So she put it in the way ‘Are you together?’ and that’s a fine piece of words. That would be a fine medical approach of asking. When people ask you ‘Are you married?’ or ‘How long have you been together?’, when people were genuinely interested, that was a positive experience’.* Aoife, bisexual woman in her 40s

Asking about relationship status was advocated by participants to ensure relationships were recognised, and shift responsibility away from the patient and partner to disclose this themselves.

*‘I don’t know, if they asked her or if she told them, but definitely, they should have a conversation about it when the patient first goes in. Ask them, ask them who the visitors are gonna be, who they are, what their relationship is and then they know’.* Lorraine, same-sex attracted, non-binary person in their 50s

#### b. Unspoken acceptance

Participants described achieving unspoken acceptance both actively and passively.

*Active positioning*: Despite being in a legally recognised relationship, many participants described a preference for unspoken acceptance. For some participants this active decision was framed positively. They talked about LGBT+ relationships being normalised, thus removing the need to disclose anything.

*‘There was no ‘Here is Anthony and [Partner) the gay couple’, it was just ‘Here is Anthony and [Partner]’ as if you’d say ‘Here’s James and Sarah’. [. . .] It was totally normalised, which was very refreshing actually. Because, you know, in the hospital environment you would think you might come across a nurse or something who looked at you sideways [. . .] In fact, almost the reverse, they were drawn to [Partner] like moths to a flame, they just loved [Partner]’.* Anthony, gay man in his 50s

However, for others this decision was framed more negatively and related to fears or experiences of discrimination, discomfort talking about sexual orientation, or sometimes simply a preference for privacy.

*‘I’m sort of a very private person [. . .] I don’t like being, being called ‘I am [Partner]’s husband’ or whatever vice versa. Erm I don’t think it, it fits me. I don’t mind other people using it but not me [. . .] I prefer friend or, or partner if, if they want to say, but not husband’.* Paulo, gay man in his 60s

Others described the decision more neutrally, or in terms of necessity. They described a need for the patient rather than the couple to be the focus of care, with any disclosure about their relationship not being a priority.

*‘You really don’t give a shit about yourself and what you have to do because your needs are so subservient to the needs of your partner. But if you were in the closet or very insecure about your sexuality then I can imagine that would be a much harder thing to deal with. Not because people were treating you badly but because it comes up so often, ‘cause you have to keep saying who you are’.* Stuart, gay man in his 40s

*Passive Positioning*: There were also examples of passive adoption of unspoken acceptance. For some this was due to a preference for not talking about the nature of their relationship, and therefore allowing their relationship to be presumed, and not initiating discussions themselves.

*‘Yeah, well it is something you never talked about [. . .] I mean it’s a different, different world today. It, it seems to be erm, compulsory now to talk about it, but in our day it just, it was never even mentioned. [INTERVIEWER: Like it was more implicit, like implied rather than spoken?] [. . .] Well not even implied, it just was never even discussed so yeah’.* Robert, man bereaved of a male partner in his 70s

For others this passive positioning came about more due to lack of communication and engagement from healthcare professionals resulting in the partner being overlooked.

*‘They never asked [. . .] I was with him and that’s it [. . .] I didn’t feel that they were curious or they, they want to know about me. But I, of course I was present and then of course some, some of them probably guessed, yeah we, we were a couple. But err, nobody asked directly’.* Paulo, gay man in his 60s

Others however talked about this more positively in relation to social support. Supportive behaviours from those around them were more important than explicit acknowledgement of the nature of the relationship itself.

*‘My neighbour next door [. . .]I’ve never actually said to him that we’re a gay couple, but he had kind of skimmed over the fact that we’re two women living together [. . .] I did tell him that she’d gone into [the hospice]. A few weeks later after she died, he asked me [. . .] ‘How is your friend?’, you know? [. . .] So I just said ‘She died’ and he reacted lovely, every time he sees me he says ‘Are you doing alright?’ and ‘How are you?’ [. . .] So yeah, he’s kind of aware and he’s been very nice’*. Aoife, bisexual woman in her 40s

However, on some occasions the decision not to be explicit about the relationship resulted in incorrect assumptions about the relationship, which added an additional layer of embarrassment and potential offence.

*‘I don’t think anybody ever said ‘Is this your partner?’ [. . .] I think a lot of people would have assumed he was my father or friend or brother or something rather than partner, just because that’s the way lots of people think. I mean if someone just says ‘What’s your relationship with [Partner]?’ then that is perfectly fine [. . .] It’s just a practical question. [. . .] I only had it a couple of times but it’s always better to ask a question than to make an assumed answer that would be wrong cause then you are at risk of causing offence’’*. Stuart, gay man in his 40s

#### c. Invisibility

Participants provided examples of their relationship not being accepted or spoken about, but only described this invisibility in the context of this being intentional (i.e. achieved actively).

*Active Positioning*: Despite the implications for impaired access to support, several participants chose to maintain invisibility within certain social spheres. Reasons for this included historical experiences of the need for caution around disclosure of relationships, a preference not to talk about relationships, and fears of discrimination. Some participants talked about not feeling comfortable to be disclose their relationships at work, and therefore their employers and colleagues being unaware of their bereavement.

*‘People at work, you go for a drink and you talk about subjects and some questions you try to avoid [. . .] People are quite erm, careful about what they’re asking. They don’t want to ask you too much. [INTERVIEWER: were you able to get any time off or anything to help you? [. . .]] When my mother died, I took bereavement leave because she was[. . .] acknowledged at work. Erm but when my partner died I just took time off’.* Alexander, gay man in his 40s

Others talked about cultural rejection of same-gender relationships. Individuals chose to keep their relationship hidden from family, in order to retain their relationship with them.

*‘I couldn’t phone his, him directly, I was phoning our friend [. . .] Where my family is living in Iran [. . .] they can’t get a signal, I had to climb a hill to make a phone call or receive a phone call. So when he died, then our friend who was looking after him, he tried to phone but he couldn’t [. . .] So, it was, he died, and I knew on, a day later. It was very difficult for me, erm because I was there and no one knew my situation here so I had to hide all my emotions, my feelings [. . .] That was the most difficult part of it’*. Jason, gay man in his 40s

#### d. Overt exclusion

Participants provided examples of their relationship not being accepted when spoken about, but only described this overt exclusion in the context of this being non-intentional (i.e. achieved passively). In these situations, the participant had disclosed their relationship, however the response from the individual forced them into a position of overt exclusion, thus passively achieved.

*Passive Positioning*: Participants described experiences of overt exclusion following disclosure due to bureaucracy (such as the requirement to use birth name of deceased transgender partners). However, they also described overt exclusion enacted by individuals aware of but refusing to recognise the nature of their relationship.

*‘I called the hospital and [. . .] they said to me [. . .] ‘We don’t know who you are and we’re not answering any questions [. . .] You need to call her sister because we’re telling her everything’ [. . .] I just felt like in intensive care they should have been a lot more understanding. [. . .] I said to them ‘I’m her next of kin on record. Look on her, her err, file’ and [. . .] the doctor I was talking to [. . .] he said he didn’t have time to look at her file and as far as he’s concerned, her sister is her next of kin*’. Marie, bisexual woman in her 50s

## Discussion

### Main findings

LGBT+ people experience devastation at the loss of a life partner, just as captured by the literature for loss of spousal partners.^2^ This is exacerbated by additional barriers and stressors specific to their sexual orientation and gender history. Participants described lack of recognition of the depth of their loss, inappropriate questioning, disclosure of gender history against their wishes, and fears of discrimination or rejection when accessing bereavement support. There were also challenges related to the intersection between their LGBT+ identities and other aspects of their culture and identity. Our findings in relation to disclosure of relationship status provide some primary evidence to support the Acceptance-Disclosure model of LGBT+ bereavement.^21^ However, for one domain, overt exclusion, we found examples only of passive rather than active positioning, and this finding helps in the interpretation of the model. We posit that it is the action of exclusion by another individual that forces the LGBT+ person into the position of overt exclusion, thus removing their source of agency.

### What this study adds

These findings build on previous theories on the concepts of disenfranchised grief^27,33^ and loss to provide new perspectives. Rather than disenfranchisement being binary in nature, individuals talked about a continuum of support and disenfranchisement within their social networks. For example, one individual might describe their relationship and bereavement being known about and acknowledged by friends, not openly discussed with neighbours, and unknown or unacknowledged by biological family and employers. This demonstrates the dynamic nature of disclosure preferences, and the importance of understanding these in order to best support individuals pre- and post-bereavement. Of particular note in relation to the Acceptance-Disclosure model of LGBT+ bereavement was the role of agency and choice in decisions regarding disclosure of identities, and the decision for some LGBT+ individuals to actively choose invisibility to retain relationships within certain social groups. This theory serves as a valuable tool for professionals caring for LGBT+ individuals peri-bereavement. However, it has relevance throughout the lifecourse for many LGBT+ people and their significant others, where decisions regarding disclosure of identities are ever present, due to the legacy of a lifetime of exclusion and discrimination.^34^ It helps explain the decisions individuals make about disclosure of their relationships and identities, the role agency plays in this process, and the implications of these on their access to bereavement support. As such, it may also have utility for individuals from other minoritised and marginalised groups whose relationships flout cultural or societal norms, and whose social support resources may be reduced.

These findings also challenge the current discourse and policy drive to mandate sexual orientation and gender identity monitoring.^35,36^ Participants were universal in their agreement of the importance of understanding who matters to the patient, and who is next of kin. However, many individuals did not feel their specific LGBT+ identity was relevant or found questions about the nature of their relationship confrontational. This view was held by both older and younger participants. The arc of legal and social change (from criminalisation and discrimination, through legalisation, to acceptance and protection) seems to have led some individuals back to a preference for unspoken acceptance. Older participants who had experienced relationships prior to legalisation of same-gender relationships were habituated into not talking about their relationships, whereas younger individuals described experiences of LGBT+ relationships and identities being normalised, obviating the need to talk about them. This finding highlights the important role of implementation research for LGBT+ monitoring, and the contribution required by both services and individuals to enable inclusive practice that is person centred.^23,24^ Service level indicators of inclusivity, and sensitive inclusive communication are key. Through using neutral language, avoiding assumptions, and being aware of non-verbal communication and the environment in which discussions are being held, professionals can offer individuals the choice and opportunity to share information about relationships and identities in line with their preferences.^24^ This in turn enables LGBT+ people to feel supported, included, safe and confident to access health and social care when they need it.^37^ Drawing on the findings of the present study five recommendations for inclusive care of LGBT+ people facing bereavement are presented (see Table 2).

**Table 2.** Recommendations for inclusive care of LGBT+ people facing bereavement.

**1.** Avoid making assumptions about individuals and relationships, because incorrect assumptions can be damaging, particularly in bereavement. **2.** Use the relationship label(s) (e.g. partner) and pronouns (e.g. she/her) that individuals themselves use, and ask if you do not know. **3.** Explore sources of personal and social support by asking: Who in their lives is important to them? Who is aware of their bereavement, and the nature of that loss? This information will help you to understand the true nature of support available from family, friends, social networks and employers, and also ensure you do not inadvertently disclose their relationship to others against their wishes. **4.** Be cognisant and respectful of the hesitancy and fears individuals may have around accessing formal bereavement support. **5.** Facilitate connections with local bereavement support services, and make the first introduction if required, to allay fears and enable individuals to access the support they need.

### Strengths and limitations

This study purposively sampled individuals who had responded to a national population-based post-bereavement survey. This enabled recruitment of a more diverse sample of participants than is common in LGBT+ research. Our data included experiences of two transgender participants, three people bereaved of a transgender partner, and individuals from minority ethnic and religious groups. The diversity of this sample will enable greater transferability of the findings. One limitation of our sampling was that all individuals were bereaved of a civil partner or spouse. It could be argued that these individuals are more likely to be well supported in bereavement, because of their legally recognised relationship. Further research including those for whom the experience of invisibility predominates, or who experience internalised stigma, would increase transferability and resonance to wider groups, and give further understanding as to how agency (active and passive positioning) contributes to the experience and disclosure preferences for these individuals.

## Conclusions

LGBT+ bereaved people face additional barriers and stressors in bereavement due to legal and financial issues, and sub-optimal support due to fears regarding disclosure of their relationship and bereavement within certain groups. Not all LGBT+ people want to talk directly about their identity or the nature of their relationship. Sensitive communication is required to build trust, and to enable discussions peri-bereavement to ensure support is in place. Particular consideration is needed for those who may be isolated or disenfranchised in their grief due to intersections related to their culture or ethnicity, and for people experiencing damaging posthumous disclosures of gender history. The legal requirements currently negate transgender inclusive care. Further work is needed to ensure bereavement support is culturally competent, and to consider how the Acceptance-Disclosure Model could inform support strategies for other potentially isolated or disenfranchised groups.

## Supplemental Material

sj-pdf-1-pmj-10.1177_02692163221138620 – Supplemental material for LGBT+ partner bereavement and appraisal of the Acceptance-Disclosure Model of LGBT+ bereavement: A qualitative interview study Click here for additional data file. Supplemental material, sj-pdf-1-pmj-10.1177_02692163221138620 for LGBT+ partner bereavement and appraisal of the Acceptance-Disclosure Model of LGBT+ bereavement: A qualitative interview study by Katherine Bristowe, Liadh Timmins, Debbie Braybrook, Steve Marshall, Alexandra Pitman, Katherine Johnson, Elizabeth Day, Paul Clift, Ruth Rose, Deokhee Yi, Peihan Yu, Wei Gao, Anna Roach, Kathryn Almack, Michael King and Richard Harding in Palliative Medicine
