# Supplementary material for: LGBT+ partner bereavement and appraisal of the Acceptance-Disclosure Model of LGBT+ bereavement: A qualitative interview study
Source: Palliat Med. 2022 Nov 25;37(2):221–34. doi: 10.1177/02692163221138620 (PMC9896255; doi:10.1177/02692163221138620)
Supplement: sj-pdf-1-pmj-10.1177_02692163221138620 – Supplemental material for LGBT+ partner bereavement and appraisal of the Acceptance-Disclosure Model of LGBT+ bereavement: A qualitative interview study [file sj-pdf-1-pmj-10.1177_02692163221138620.pdf]

ACCESSCARE B: TOPIC GUIDE
FOR BEREAVED PARTNERS

Introductory Statement (for all participants):

Thank you for agreeing to participate in a research interview. We are researchers from King's College London, and we are working on a research project which is looking to improve the care that people receive who have recently lost a partner or spouse. As we will be asking you about your identity, your home life, and the care that you and your partner received, there may be some questions that you find sensitive. If there are any questions you would rather not answer then we can move on, or if you want to stop the interview at any time, just let me know. Everything you say in this interview will be confidential. If we use quotes for our research, we will make sure that you cannot be identified from the quote, by replacing names, places and any other identifiable information. Do you have any questions before we start?

Topic guide:

Demographic information (for all participants):

- Age (for decedent and bereaved caregiver)
- Gender / Gender identity (for decedent and bereaved caregiver)
- Sexuality (for decedent and bereaved caregiver)
- Cultural identity or ethnicity (for decedent and bereaved caregiver)
- Relationship to decedent
- Primary diagnosis
- Time since diagnosis
- Care settings

Illness History and Experience (for all participants):

- Can we please start by telling me about X's illness, when did he/she first became unwell?
- Can you tell me where X received care for his/her illness? (PROMPT: Primary secondary tertiary care)
- What were X's main needs and problems? (PROMPT: physical, psychological, social spiritual)

Involvement of Partner/Caregiver (for all participants):

- What sort of a caregiving role did you have with X during his/her illness?
- Did you tend to go to clinical meetings and appointments with X?
- Did he/she want you to be present?
- Who introduced you to staff, and how were you introduced?
- How welcome did you feel, from the health care providers?
- How did they acknowledge you?
- How was the experience for you?
- Would you have liked it to be any different? How?
- Did they ever ask about you about your needs?
- Do you feel you had enough support at that time?

- What forms of additional support would have been useful for you?
- Were there any questions you would have liked to have asked the healthcare team but didn’t or couldn’t?
- Were there any questions you felt the health care providers didn’t ask you?

Exploration of Sexual Identity for (LGB participants):

- Did X’s health care providers, know his/her sexual identity?
- YES….how?
- NO….why?
- Is this how he/she preferred it to be?
- How did he/she feel about being asked directly about his/her sexual identity?
- How did he/she like his/her sexual identity to be acknowledged and referred to by health care providers?
- How about for you, how do you feel about sharing your sexual identity?
    - Prompt: with friends, family, colleagues, acquaintances
- How do you feel about being asked directly about your sexual identity?
- As someone who identified as Y, in what ways do you think X’s experience of illness might have been different to others?
- How about X’s experience of care, how might that have been different?
- How about for you, do you feel your experience may have been different because of X’s sexual identity / your relationship?

Communicating and Sexual Identity (for partners of LGB people only):

- Do you feel there are things that make it easier/less easy to talk to health care providers about sexual identity?
- PROMPT: communicative, environmental, societal, institutional
- Are there phrases or words that the health care providers have used that made you and X feel more able to talk about X’s sexual identity?
- How about in terms of the way they talk to you – their manner, and the non-verbal cues?
- Do you find the health care providers use the same words to describe sexual identity as you and X?
- What did health care providers do well? What could have been better?
- Can you tell me about when it hasn’t been handled well?
- Are there phrases or words used by the health care providers that made you and X feel uncomfortable about sharing his/her sexual identity?
- Were there times when their manner or non-verbal cues stopped X from sharing his/her identity? How?

Exploration of Gender Identity (for partners of trans people only):

- Did X’s health care providers, know his/her gender identity/gender history?
- YES….how?
- NO….why?
- Is this how he/she preferred it to be?
- How did he/she feel about being asked directly about his/her gender identity?
- How did he/she like his/her gender identity to be acknowledged and referred to?

- As someone who identified as Y, in what ways do you think X's experience of illness might have been different to others?
- How about X's experience of care, how might that have been different?
- How about for you, do you feel your experience may have been different because of X's gender identity?

Communicating and Gender Identity (for partners of trans people only):

- Do you feel there are things that make it easier/less easy to talk to health care providers about gender identity? (PROMPT: communicative, environmental, societal, institutional)
- Are there phrases or words that the health care providers have used that made you and X feel more able to talk about X's gender identity?
- How about in terms of the way they talk to you – their manner, and the non-verbal cues?
- Do you find the health care providers use the same words to describe gender identity as you and X?
- Can you tell me about when it hasn't been handled well?
- Are there phrases or words used by the health care providers that made you and X feel uncomfortable about sharing his/her gender identity?
- Were there times when their manner or non-verbal cues stopped X from sharing his/her identity? How?

Support Structures (for all participants):

- Where did X get his/her support from?
    - PROMPT: Family, communities, institutional
- Were they known to X's health care team? How/why?
- Who would you say was his/her main source of care?
    - PROMPT: outside of professional services like doctors nurses and social worker)
    - PROMPT: biological vs chosen family and networks

Planning for Future Care:

- Thinking back, did X ever talk about who he/she would want to be involved if his/her health changed and decisions needed to be made?
- Did he/she have a chance to talk to someone about this?
- Did this happen? Were there any problems?

Reflections and Recommendations (for partners of LGB people only):

- Thinking about X, his/her life, and what mattered to him/her, do you think his/her sexual identity should have been part of discussions with the healthcare teams?
    - PROMPT: Why? How?
- Since X had this diagnosis, did you or he/she ever feel that he/she was treated differently or unfairly because of his/her sexual identify?

Reflections and Recommendations (for partners of trans people only):

- Thinking about X, his/her life, and what mattered to him/her, do you think his/her gender history should have been part of discussions with the healthcare teams?
    - PROMPT: Why? How?

- Since X had this diagnosis, did you or he/she ever feel that he/she was treated differently or unfairly because of his/her sexual gender history?

Funeral and bereavement experiences:

- Can you tell me about X's funeral?
- Who was involved in the arrangements?
- Had you and X discussed his/her funeral?
- Was the service what he/she would have wanted?
- Was the service as you wanted it to be?
- PROMPT: memorialisation and property/legal issues, if appropriate

Sources of support:

- Since X died, who has been your main source of support?
  - PROMPT: biological family, chosen family, friends, work colleagues, partner's family
- What support have you received from those around you?
  - PROMPT: biological family, chosen family, friends, work colleagues, partner's family
- How about from neighbours and other social networks, are they aware of your loss?
- Are you employers aware of your loss? How have you been supported by your employer and work colleagues?
- As someone who identifies as LGB, do you think the support you have received has been any different to the support heterosexual people might receive (partners of LGB people only)?
- As the partner of someone who identifies as trans, do you think the support you have received has been any different to the support received by partners of cisgender people (partners of trans people only)?

- Have you sought any professional support since your bereavement?
  - PROMPT: GP, counselling, bereavement services, psychological/psychiatric services
- If so, how did you access the support?
- Were you referred or did you seek it yourself?
- What was it like to attend the support group/counselling?
- If not, was there anything in particular that stopped you from accessing support?
  - PROMPT: sexual orientation, gender history (partners of LGBT people only)
- If not, do you think you might access some support in the future?

Recommendations and reflections:

- Thinking about your experiences, what has been the most valuable source of support for you since your bereavement
  - PROMPT: close networks, societal and professional.
- What recommendations would you make to health and social care professionals supporting people who have lost a partner?
  - PROMPT: discussion about significant others, communication, services
- Thinking back to when you were first bereaved, what recommendations would you make to someone who has recently lost their partner?
- Is there anything further you would like to add?
